# Long Noncoding RNAs as Novel Biomarkers Have a Promising Future in Cancer Diagnostics

**DOI:** 10.1155/2016/9085195

**Published:** 2016-04-10

**Authors:** Ting Shi, Ge Gao, Yingli Cao

**Affiliations:** Faculty of Laboratory Medicine, Xiangya Medical College, Central South University, Changsha, Hunan 41001, China

## Abstract

Cancers have a high mortality rate due to lack of suitable specific early diagnosis tumor biomarkers. Emerging evidence is accumulating that lncRNAs (long noncoding RNAs) are involved in tumorigenesis, tumor cells proliferation, invasion, migration, apoptosis, and angiogenesis. Furthermore, extracellular lncRNAs can circulate in body fluids; they can be detected and strongly resist RNases. Many researchers have found that lncRNAs could be good candidates for tumor biomarkers and possessed high specificity, high sensitivity, and noninvasive characteristics. In this review, we summarize the detection methods and possible sources of circulating lncRNAs and outline the biological functions and expression level of the most significant lncRNAs in tissues, cell lines, and body fluids (whole blood, plasma, urine, gastric juice, and saliva) of different kinds of tumors. We evaluate the diagnostic performance of lncRNAs as tumor biomarkers. However, the biological functions and the mechanisms of circulating lncRNAs secretion have not been fully understood. The uniform normalization protocol of sample collection, lncRNAs extraction, endogenous control selection, quality assessment, and quantitative data analysis has not been established. Therefore, we put forward some recommendations that might be investigated in the future if we want to adopt lncRNAs in clinical practice.

## 1. Introduction

The human genome is composed of large and complex nucleotide sequences, which can produce more than 100000 proteins through transcription and translation. Only 2% of genomic transcripts have protein-coding ability; the remaining 98% do not have protein encoding function [1] but exert an enormous function on the process of cell biology; these kinds of RNAs are called ncRNAs (noncoding RNAs). ncRNAs are divided into short noncoding RNAs (20–200 nt) and long noncoding RNAs (200 nt–10 kb). lncRNAs account for more than 80% of ncRNAs [2, 3]. In recent years ncRNAs have attracted increasing attention of researchers, as they have found ncRNAs play a crucial role in regulating genes transcription process. miRNAs, as a member of short noncoding RNAs, participate in cellular process through targeting hundreds of mRNAs and they serve as oncogenes or tumor suppressor genes in cancer to promote or inhibit the initiation and progression of cancer [4]. Furthermore, they can be detected in body fluids such as blood, urine, breast milk, cerebrospinal fluids, and bronchial lavage [5–7] which endow them with the potential to be developed as tumor biomarkers and contribute to diagnosis, prognosis, and classification of cancers [8, 9]. However, there are no consistent results between the studies that deem the microRNAs as biomarkers [10]. Therefore, seeking alternative and complementary biomarkers is urgently needed.

Concerning the potential of being tumor biomarkers of miRNAs and the proportion of lncRNAs in ncRNA, researchers speculated lncRNAs may be promising alternatives and then transferred their focus on lncRNAs which were previously regarded as junk genes. Mercer et al. [11] found the expression of lncRNAs had temporal and tissue specificity. The genes coding lncRNAs either overlap with protein-coding and noncoding genes or are scattered between them. This kind of localization is beneficial for them to regulate the transcription of adjacent genes [12]. Ectopic expression of lncRNAs is responsible for tumorigenesis. Firstly, lncRNAs regulate some oncogenes and tumor suppressor genes both at transcriptional and posttranscriptional levels, ultimately affecting the proliferation, apoptosis, invasion, migration, and metastasis of tumor cells [13, 14]. Secondly, lncRNAs regulate chromatin remodeling and are essential to the integrity of nuclear structure [15]. Thirdly, lncRNAs can also induce epithelial-to-mesenchymal transition (EMT) via the PI3K-AKT and Wnt/*β*-catenin pathway [16, 17] to promote cancer metastasis. Although the concrete functional roles of lncRNAs in cancer are unclear, we are positive about their future applications in human tumors.

Despite ribonucleases abundantly existing in body fluids, researchers have found lncRNAs could be detected and could resist ribonucleases degradation activities. Detection of circulating lncRNAs in body fluids can be a novel noninvasive method and can be used in the assessment of cancers at the following aspects: (1) distinguishing tumor patients from healthy people at early stage with high sensitivity and specificity; (2) predicting the prognosis of tumor patients; (3) predicting the risk of tumor metastasis and tumor recurrence after surgical operation; (4) as an assessment index to evaluate whether operation is successful or not. Above all, utilizing circulating lncRNAs as tumor biomarkers has vital significances for clinical research.

## 2. Detection Methods and Sources of Circulating lncRNAs

The stability of lncRNAs in body fluids of tumor patients has been explored in limited studies. Some researchers have found lncRNAs remained stable in plasma even under oppressive conditions such as multiple cycles of freeze-thaw, incubation at 45°C for 24 hours, and incubation at room temperature for up to 24 h [18]. To choose the suitable vacutainer tubes to collect specimens, Ren et al. [19] compared the level of lncRNAs among heparin plasma, EDTA plasma, and serum. They found EDTA plasma and serum both maintained the stability of lncRNAs effectively, whereas the expression level of lncRNAs in heparin plasma had a significant decline. Thus we can use EDTA vacutainer tubes or the tube without anticoagulant to collect blood specimen for analyzing the expression of plasma lncRNAs.

Regarding the detection method of lncRNAs, quantitative real-time PCR is considered to be the gold standard for quantitative expression analysis of lncRNAs. However, in the aspects of extraction methods and kits, endogenous controls, and quantification methods, there exists no unified standard. Quantification methods are divided into relative quantification and absolute quantification methods. In relative quantification methods, the levels of lncRNAs are determined by the ΔΔC_T_ method. In order to produce reliable results, the endogenous controls for normalization should be chosen correctly and should be tested prior to application. Weber et al. [20] measured the potential reference genes GADPH, HRPT1, and RPLP0 in NSCLC patients and normal controls; RPLP0 was excluded from the reference genes due to its different expression between NSCLC patients and normal controls. Although RPLP0 is a suitable reference gene for analyses in NSCLC tissues [21], it is not feasible as an endogenous control in human blood. Considering that there has been no consensus on the stable and suitable endogenous controls, some researchers used absolute quantification method, and the level of lncRNAs is determined by the standard curve constructed with reference standards [22].

**Figure 1:**
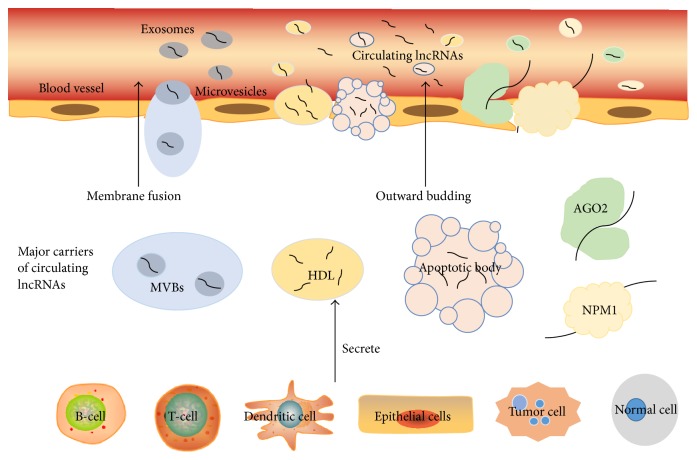
The origin of circulating lncRNAs and several manners of circulating lncRNAs encapsulation.

The molecule mechanisms underlying circulating lncRNAs secretion and transport to the extracellular environment have not been clear and have been only elucidated in a few studies. Some researchers speculated the secretion of lncRNAs may be in the same manner as miRNA. According to the relevant studies, lncRNAs may secrete in three manners as shown in Figure 1: (1) extracellular RNAs package themselves into membrane vesicles to secrete and resist RNase, such as exosomes and microvesicles. Exosomes and microvesicles are 50 to 100 nm long in diameter lipoprotein vesicles and are formed by inward budding of multivesicular bodies (MVBs) through the endocytic pathway. Then through the fusion of MVBs with plasma membrane, exosomes secreted consistently [23]. Initially, exosomes seemed as discarded membrane proteins without any biological functions [24]. However, more recent studies have shown that exosomes and microvesicles that encapsulate both RNA and proteins can be messengers to transfer information via regulating transcription and translation processes. Huang et al. [25] did a deep-sequence to characterize human plasma-derived exosomal RNAs and finally detected that lncRNAs accounted for 3.36% of all exosomal RNAs sequences. Another study showed that there were no significant differences of lncRNA levels in plasma and exosomes. These results indicated lncRNAs mainly exist in exosomes and exosomes are the main protector for plasma lncRNAs [26]. (2) Extracellular RNAs are released by tumor tissues and cells. Ren et al. [19] identified lncRNA levels drastically increased in plasma due to the presence of subcutaneous xenograft cancer. Moreover, the expression level of plasma lncRNAs was decreased from the preoperative patients to the postoperative patients. This suggested lncRNAs were derived from the tumor cells and could enter the circulation. Besides circulating and primary tumor cells, circulating lncRNAs have multiple sources, such as cancer-adjacent normal cells, immune cells, and other blood cells [18, 26]. (3) Extracellular RNAs encapsulate themselves into high density lipoprotein (HDL) or apoptosis bodies or associated with protein complexes. The most typical protein complexes are Argonaute- (Ago-) miRNA complex [27] and nucleophosmin 1- (NPM1-) miRNA complex [28].

The biological functions of circulating lncRNAs and the possibility of lncRNAs becoming disease-specific noninvasion biomarkers are poorly understood. The mechanisms regulating circulating lncRNAs expression remain unclear; however, if we want to find out the relationship between circulating lncRNAs expression level and cancers, we must break through these issues in the future.

## 3. MALAT1 in Cancer Tissues, Cell Lines, and Body Fluids

### 3.1. Lung Cancer (LC)

Metastasis-associated lung adenocarcinoma transcript 1 (MALAT1) was first studied in the metastatic lung adenocarcinoma patients. In order to find which genes are associated with LC metastasis, Ji et al. [29] utilized subtractive hybridization method to analyze different gene expressions between metastatic and nonmetastatic lung tumor tissues. They identified MALAT1 expressed threefold higher in metastatic tumor tissues. In the early stage of non-small cell lung cancer (NSCLC), patients with high expression level of MALAT1 had a high risk of subsequent metastasis and poor prognostic. Schmidt et al. [30] studied the biological function of MALAT1 at a cellular level which found high MALAT1 expression in mouse fibroblast cell line (NIH3T3) increased its migratory and invasive capacity, while low MALAT1 expression in A549 cell line impaired its formation and growth. To further elucidate the mechanism of MALAT1 getting involved in metastatic process, Tano et al. [31] knocked down MALAT1 by short-interfering RNA (siRNA) in A549 cells, simultaneously observing some motility-associated genes such as* CTHRC1* (collagen triple helix repeat containing),* CCT4* (chaperonin-containing tailless complex polypeptide, subunit 4), and* ROD1* (regulator of differentiation 1). Expression levels were downregulated at the mRNA level whereas* AIM1* (melanoma 1),* LAYN* (layilin), and* HMMR* (hyaluronan-mediated motility receptor) were reduced at both pre-mRNA and mature mRNA levels. This suggested MALAT1 modulated genes expression at transcriptional and posttranscriptional levels except for regulating splicing to enhance cell migration. Moreover, MALAT1 could be involved in EMT process to promote tumor metastasis [32]. Gutschner et al. [33] utilized Zinc Finger Nucleases (ZFN) to build loss of function cells. After injection of MALAT1 knock-out cells in the nude mice, the number of tumor nodules was reduced. Furthermore, tumor volume and tumor weight decreased after treatment with MALAT1 antisense oligonucleotides (ASO). All these indicated that MALAT1 could be an effective therapeutic target in lung cancer in the future.

MALAT1 not only has a crucial significance in predicting metastasis risk and may be a promising therapeutic target in treating lung cancer, but also can be a diagnostic biomarker detectable in blood to screen lung cancer. Guo et al. [34] analyzed the expression level of MALAT1 in 105 lung cancer patients and 65 healthy persons' whole blood by quantitative polymerase chain reaction (qPCR). Surprisingly, LC patients had lower expression of MALAT1 than healthy subjects in whole blood which was contrary to the high expression of MALAT1 in lung cancer tissues. But one common thing in tissues and whole blood was that metastatic lung cancer patients had stronger expression of MALAT1 than nonmetastatic lung cancer patients. Furthermore, compared with lymph node and pleura metastasis, bone and brain metastasis had high expression of MALAT1. In the cellular fraction of peripheral human blood, MALAT1 as a biomarker of screening NSCLC exhibited low sensitivity of 56% and high specificity of 96%, which demonstrated MALAT1 cannot be an independent but a complementary biomarker to diagnose NSCLC [20].

### 3.2. Prostate Cancer (PCa)

The expression level of MALAT1 in PCa tissues was first investigated by Ren et al. [35]. They used RNA-seq method to analyze the expression of lncRNAs in 14 pairs of PCa and adjacent normal tissues. The results showed MALAT1 was overexpressed in PCa tissues and high expression of MALAT1 had close relationships with high Gleason score, prostate specific antigen (PSA), and tumor-node-metastasis (TNM) stage. Then they studied the biological function of MALAT1 in PCa at a cellular level.* In vitro*, they silenced MALAT1 in prostate cell lines 22Rv1 and LNCaP that ultimately inhibited the migratory and invasive capacity of cancer cells and made cell cycle be arrested in G0/G1 phases [36].* In vivo*, the growth of tumor xenografts became slower and metastatic rate was reduced after injecting MALAT1 knock-out cells in the nude mice. Additionally, the survival time of tumor mice was prolonged [36]. Above all, MALAT1 can be an interesting therapeutic target for PCa.

Unlike the low expression of MALAT1 in the whole blood of lung cancer patients, the expression of MALAT1 derived miniRNA (MD miniRNA) was elevated in plasma of PCa patients, which was consistent with MALAT1 expression in PCa tissues. PSA is a prostate-specific biomarker. As a diagnostic marker, PSA alone could be very sensitive but with low specificity which finally brings about lots of unnecessary biopsies and repeated biopsies [37]. Compared to PSA, MD miniRNA was more effective plasma-based biomarkers to diagnose PCa from non-PCa. MD miniRNA was also more effective at distinguishing positive prostate biopsies and negative prostate biopsies (AUC 0.841) than PSA (AUC 0.708) [19]. MD miniRNA was very helpful to predict prostate biopsy outcome. Furthermore, combined PSA and MD miniRNA could improve the sensitivity and specificity of diagnosis [19]. Besides plasma, Wang et al. [38] evaluated the diagnostic value of MALAT1 in urine. They observed that, with the level of PSA in gray zone (4–10 ng/mL), setting urinary MALAT1 score cut-off of 95 had higher sensitivity and specificity than the %  fPSA (percentage of free PSA), which is the conventional reliable index for PCs diagnosis [38]. Furthermore, using the probability threshold of 25%, more cancer could be detected and unnecessary and repeated biopsies could be avoided 30.2%–46.5% in PSA 4–10 ng/mL cohorts [38].

## 4. H19 in Cancer Tissues, Cell Lines, and Body Fluids

### 4.1. Gastric Cancer (GC)

The lncRNA expression profile in GC was uncovered by Song et al. [39]. They recruited GC tissues and noncancerous tissues to do lncRNA microarray analysis, which found the gene H19 expressed up to 8.91-fold higher in GC tissues. Initially H19 was considered as tumor suppressor gene because of its role in inhibiting tumorigenicity [40, 41], but recent researchers have identified that H19 can play a role as an oncogene. Li et al. [42] have observed that the proliferative, invasive, and migratory abilities of cells were repressed after the knocking down of H19, whereas, after subcutaneously injecting SGC7901 cells in nude mice, which were transfected with H19, the rate of tumor growth, tumor size, tumor weight, and the number of metastasis nodules were increased. The concrete regulation mechanisms of H19 as an oncogene include regulating tumor suppressor gene p53 [43] and H19/miRNA/tumor suppressor gene axis. Yang et al. [44] found the activity of p53 significantly decreased when H19 and p53-responsive reporter plasmids were cotransfected into AGS human gastric cell lines. Zhuang et al. [45] transfected MGC803 cells with siRNA-H19 and miR-675 mimics simultaneously, and the overexpression of miR-675 restored siRNA-H19-induced inhibition. In treated AGS cells with pcDNA-H19 and anti-miR-675, anti-miR-675 rescues pcDNA-H19-induced promotion of GC cell proliferation. These results demonstrated that H19 regulated GC cells proliferation phenotype through miR675. Through several algorithms, researchers found miR-675 regulated cell proliferation of GC cells by targeting the tumor suppression gene Runt Domain Transcription Factor 1 (RUNX1) and consisted of the H19/miR675/RUNX1 axis pathway [45]. Besides RUNX1, ISM1 is another target protein of H19 [42]. All these molecules can be promising therapeutic targets of GC.

Based on the studies of H19 in GC tissue, researchers were interested in whether H19 can apply in clinical practices as a novel noninvasion biomarker to screen gastric biomarkers. Zhou et al. [22] selected 8 lncRNAs in which their aberrant expression in GC tissue was previously found and validated their expression in plasma. They found that, among 8 lncRNAs, HOTAIR (Homeobox protein transcript antisense RNA), MALAT1, and H19 were highly expressed in GC plasma. However, another study found the opposite results that HOTAIR and MALAT1 had no significant difference in expression of patients and controls. Moreover, this research identified that MALAT1 was not so stable in the plasma, which was contradicted by MD miniRNA's stability in PCa plasma even in a harsh environment [19]. H19 is highly expressed in GC plasma and could serve as a promising biomarker of GC due to its high diagnostic power for detection of GC (AUC 0.838; specificity 72.9%; sensitivity 82.9%). H19 could also screen for early stage of GC (AUC 0.877; specificity 80.1%; sensitivity 85.5%), which was more effective than the conventional biomarkers such as carcinoembryonic antigen (CEA) and carbohydrate antigen 199 (CA199) [22].

## 5. HOTAIR in Cancer Tissues, Cell Lines, and Body Fluids

### 5.1. Colorectal Cancer (CRC)

HOTAIR had oncogenic properties. Its high expression in CRC tissues was closely related to vascular invasion, histological differentiation, and liver metastasis [46, 47]. Patients with high expression of HOTAIR were more likely to have tumor recurrence and poor prognosis. Furthermore, Cai et al. [48] did a meta-analysis which included 748 patients from 8 studies to evaluate the association between HOTAIR expression levels and lymph node metastasis. They identified that patients with high expression of HOTAIR had a high incidence of lymph node metastasis. The mechanisms of HOTAIR regulating the initiation and progression of CRC include relying on Polycomb Repressive Complex (PRC) complex to silence tumor suppressor genes and being involved in EMT process. HOTAIR gene is located on chromosome 12q13.13 between the HoxC11 and HoxC12 exons [49]; it can reprogram chromatin organization by binding with PRC at 5′ domain and lysergic acid diethylamide (LSD) at 3′ domain in CRC [46, 50]. PRC complex silences tumor genes at the trimethylation of histone H3 lysine 27 (H3K27) through a methylation process. PRC complex contains an enhancer of zeste homolog 2 (EZH2), SUZ12, and EED three subunits [51]. Among the three subunits, EZH2 is the core enzyme in methyl transfer process and EED regulates the specialty of EZH2 by binding to target RNA [52]. Wu et al. [47] identified that after knocking down HOTAIR in SW480 and HT29 CRC cell lines, E-cadherin as marker of epithelial cell was increased and mesenchymal cell marker vimentin was decreased. Moreover, the major proteinase MMP9, which impaired the motile ability of cells, was inhibited. All these studies indicated that HOTAIR could be an independent biomarker for CRC diagnosis.

Currently, there is only one study that focused on whether the expression level of HOTAIR in blood can serve as a potential marker for CRC prognostic [53]. This study simultaneously detected expression levels of HOTAIR in 73 CRC tissues and 84 CRC bloods. Surprisingly, their results in CRC tissues contradicted other studies that showed there were no significant differences between tumor tissues and noncancerous tissues, whereas, in blood, HOTAIR was upregulated and expressed higher in left (descendent) colon tumors compared with right (ascendant) colon tumors. Furthermore, expression of HOTAIR can predict the survival time of patients. High expression of HOTAIR had high risk of death and was associated with poor prognosis. Evaluating prognostic power of HOTAIR by ROC curve (AUC 0.87)—its specificity was 67% and sensitivity was 92.5%—demonstrated HOTAIR can be a negative prognostic marker not only in tumor tissues but also in blood.

### 5.2. Oral Squamous Cell Cancer (OSCC)

The lncRNA expression profile in OSCC was first investigated by Gibb et al. [54]. In OSCC, HOTAIR was overexpressed in tumor tissues compared to normal tissues and its high expression was correlated to tumor size, clinical stage, lymphatic node metastasis, and histological differentiation [55, 56]. By knocking down HOTAIR in Tca8113 OSCC cell line, we found cells were arrested in G0/G1 phase and apoptosis process was accelerated. The proliferation rate of cell was reduced and the invasion and migration of cell were inhibited [55, 56]. Moreover, the enrichment of EZH2 and H3K27me3 was significantly decreased while the expression of E-cadherin significantly increased after knocking down HOTAIR [57, 58]. Treating Tca8113 and TSCCA cells with siEZH2 and siEZH2+siHOTAIR caused E-cadherin expression levels to increase at both mRNA and protein levels. These results elucidated HOTAIR participating in EMT process through interacting with EZH2 and H3K27me3 at the E-cadherin promoter [56]. Therefore, HOTAIR plays a critical role in the development and progression of OSCC and can be a predictive marker as well as a new therapeutic target in OSCC.

Tang et al. [59] detected lncRNA in saliva of OSCC patients. 1 mL salivary samples were collected from 9 OSCC patients and the presence of lncRNA was examined by qPCR. C_t_ < 40 was considered to be positive. Finally, they found MALAT1 was detected in all samples but had no significant difference in metastatic and nonmetastatic tissues, while HOTAIR was detected only in 5/9 patients and the patients with lymph node metastasis had higher expression of HOTAIR in saliva. All these suggest detecting lncRNA in saliva may provide clinics with a noninvasive and convenient tool to screen OSCC.

## 6. LINC00152 in Cancer Tissues, Cell Lines, and Body Fluids

### 6.1. Gastric Cancer (GC)

LINC00152 is located on chromosome 2p11.2 and its transcript length is 828 nt. There were several studies that demonstrated a novel long intergenic noncoding RNA (lincRNA), LINC00152, was overexpressed in GC tissues [60, 61]. Pang et al. [60] detected the expression levels of LINC00152 in 71 pairs of GC and adjacent normal tissues. They found LINC00152 had significantly higher expression levels in GC patients and this high expression level was correlated with tumor invasion. Its diagnostic value was evaluated by ROC curve (AUC 0.645; specificity 68.1%; sensitivity 62.5%). In addition, compared with the normal human epithelial cell line GES-1, LINC00152 expression was also higher in GC cell lines, such as BGC-823, MGC-803, and SGC-7901 [60].

Consistent with the results in GC tissues, LINC00152 levels in plasma significantly increased in both advanced GC patients and early GC patients. In addition, compared with preoperative plasma samples, the expression level of LINC00152 was elevated in postoperative plasma samples [26]. This result was opposite to other studies. The author speculated that operations may stimulate cells to release lncRNAs into the plasma. Plasma LINC00152 diagnostic value (AUC 0.675; specificity 85.2%; sensitivity 48.1%) was better than conventional markers such as CEA and CA199 [26]. Therefore, LINC00152 can be a novel blood-based biomarker to apply in clinical practices to diagnose GC. Besides plasma, gastric juice can be used in another noninvasion method for screening for GC [62]. LINC00152 could also be detected in gastric juice and its expression level was higher in the patients with GC than normal people [60]. However, the function of LINC00152 in GC is still unclear and the sample size detected in plasma was very small. Therefore further study should investigate the function of LINC00152 and expand the sample size.

## 7. Conclusions and Perspectives

In this review, the biological functions and diagnostic value of lncRNAs are assessed in Table 1. The largest troublesome problem of the treatment of various cancer is the lack of the early diagnosis approaches. Some kinds of cancers could be completely cured if the patients are diagnosed at an early stage. However, conventional tumor markers such as CEA, CA199, and CA129 have low specificity or low sensitivity which leads to clinical false negative rate and false positive rate increase. Thus, finding out more effective markers is urgently needed. lncRNAs were formerly regarded as “junk” or “noise” within genomes which have no protein-coding ability. But recently, with the development of high-throughput sequencing methods such as microarray and RNA-seq, researchers disproved previous ideas and found ectopic lncRNAs expression was involved in tumorigenesis, tumor cells proliferation, invasion, migration, apoptosis, and angiogenesis [63]. Furthermore, we are confident that lncRNAs in body fluids may serve as promising biomarkers for cancers diagnosis and prognosis. Utilizing circulating lncRNAs as biomarkers has several advantages such as the following aspects: (1) they have higher specificity and sensitivity than conventional protein-based markers. (2) They can monitor disease status dynamically such as predicting the survival time of patients and predicting the risk of tumor metastasis and recurrence. (3) Detection of lncRNAs in body fluids as a noninvasion and convenient method is easy to be clinically accepted. It can ease the pain from the biopsy, save the cost of patients, and have good repeatability in clinical practice. (4) They can be independent diagnostic biomarkers or a gorgeous addition to classical noninvasion biomarkers of cancers.

Although we found lncRNAs were detectable and stable in body fluids, the mechanisms underlying lncRNAs secretion and transport to the circulation are poorly understood. Moreover, lncRNAs biological functions in cancers are not thoroughly reported. In our review, we observed that some studies are contradicting to each other. Therefore, if we want to adopt circulating lncRNA into clinical practice, further studies should investigate the following aspects: (1) building a standardization of sample preparation such as the following: which anticoagulant should be chosen, how much volume is required for sample collecting, and how high the temperature should be to store the samples. (2) Endogenous controls of lncRNAs in body fluids and the extraction methods should be uniformed. Adopting different endogenous control means adopting different quantitative standards and different extract methods that have different extract efficiency may influence the purity and concentration of lncRNAs. (3) The standard of assessing the quality of lncRNA and the credibility of qPCR results should be more accurate and reliable. (4) The research cohort size should be bigger and have reduced selection bias as much as possible.

In summary, lncRNAs not only can be potential biomarkers for early diagnosis and prognosis of cancers but also can be critical therapeutic targets.

## Figures and Tables

**Table 1:** Aberrant lncRNAs expression in various cancers.

lncRNAs	Cancer type	Sample	Up/down	Extraction method	Normalization	Quantification method	Diagnostic performance	Reference
MALAT1	NSCLC	Tissue	Up	TRIzol Reagent (Invitrogen)	GADPH	qRT-PCR	Null	[29]
		Tissue	Up	Qiagen RNeasy Micro Kit	GAPDH	SYBR-Green qRT-PCR	Null	[30]
		Cell lines (A549, HTB53, 56, 57, 58)	Up	TRIzol Reagent [29, 31] (Invitrogen), QiagenRNeasy Micro Kit [30]	GADPH	SYBR-Green qRT-PCR	Null	[29–31]
		Peripheral whole blood	Down	AxyPrep*™* Blood Total RNA Miniprep kit	GAPDH	qRT-PCR	AUC 0.718	[34]
		Cellular fraction of peripheral human blood	Down	RiboPure BloodKit (Life Technologies)	GAPDH HPRT1	TaqMan	AUC 0.79, specificity 96%, sensitivity 56%	[20]

MALAT1	PCa	Tissue	Up	TRIzol Reagent [19] (Invitrogen)	GAPDH [35] ACTB [19]	SYBR-Green qRT-PCR	Null	[19, 35]
		Plasma	Up	mirVana PARIS Kit (Ambion)	Input amount Standard curve method	SYBR-Green qRT-PCR	AUC 0.836, specificity 84.8%, sensitivity 58.6%	[19]
		Urine	Up	TRIzol Reagent (Invitrogen)	PSA	SYBR-Green qRT-PCR	AUC (0.670 and 0.742)	[38]

H19	GC	Tissue	Up	TRIzol Reagent (Invitrogen)	ACTB	GoTaq qRT-PCR [39] SYBR-Green qRT-PCR [42, 44]	Null	[39, 42, 44]
		Tissue	Up	TRIzol Reagent (Invitrogen)	GAPDH	SYBR-Green qRT-PCR	Null	[45]
		Tissue	Up	RecoverAll Total Nucleic Acid Isolation Kit	ACTB	TaqMan	Null	[18]
		Cell lines (AGS, MGC-803, SGC-7901, SMMC-7721, HepG2, Du145, PC-3 A549)	Up/down	TRIzol Reagent (Invitrogen)	ACTB	GoTaq qRT-PCR	Null	[39]
		Cell lines (AGS, MKN45, MG-C803, SGC7901, GES-1)	Up	TRIzol Reagent (Invitrogen)	ACTB [42, 44] GAPDH [45]	SYBR-Green qRT-PCR	Null	[42, 44, 45]
		Plasma	Up	mirVana PARIS Kit (Ambion)	Relative to the level of plasma lncRNAs	TaqMan	AUC 0.640, specificity 58%, sensitivity 74%	[18]
		Plasma	Up	TRIzol Reagent (TaKaRa)	Standard curve constructed with lncRNA probes	SYBR-Green qRT-PCR	AUC 0.838, specificity 72.9%, sensitivity 82.9%	[22]

HOTAIR	CRC	Tissue	Up	ISOGEN (Nippon Gene), QIAamp DNA Micro Kit [46], AllPrep DNA/RNA Mini kit [47]	GAPDH	SYBR-Green qRT-PCR	Null	[46, 47]
		Cell lines (HT29, SW480)	Up	AllPrep DNA/RNA Mini kit	GAPDH	SYBR-Green qRT-PCR	Null	[47]
		Blood	Up	MirVana Isolation Kit (Life Technologies)	ACTB, GAPDH, HPRT, PPIA, GUS 18S rRNA,	TaqMan	AUC 0.870, specificity 92.5%, sensitivity 67%	[53]

HOTAIR	OSCC	Tissue	Up	TRIzol Reagent	GAPDH	SYBR-Green qRT-PCR	Null	[55, 56, 59]
		Cell lines (TSCCA, Tca8113)	Up	TRIzol Reagent (Invitrogen)	GAPDH	SYBR-Green qRT-PCR	Null	[56]
		Saliva	Up	TRIzol (Invitrogen)	GAPDH	SYBR-Green qRT-PCR	Null	[59]

LINC00152	GC	Tissue	Up	TRIzol (Invitrogen)	GAPDH	GoTaq qRT-PCR	Null	[60]
		Cell lines (AGS, BGC-823, MGC-803, SGC-7901)	Up	TRIzol (Invitrogen)	GAPDH	GoTaq qRT-PCR	Null	[60]
		Gastric juice	Up	TRIzol LS Reagent (Invitrogen)	GAPDH	GoTaq qRT-PCR	AUC 0.645, specificity 68.1%, sensitivity 62.5%	[60]
		Plasma	Up	TRIzol LS Reagent (Invitrogen)	GAPDH	GoTaq qRT-PCR	AUC 0.675, specificity 85.2%, sensitivity 48.1%	[26]
